# Alternative Splicing and Gene Expression Variation Underlie Population and Life History Differences in an Amphibian

**DOI:** 10.1002/ece3.72481

**Published:** 2025-11-16

**Authors:** Juntao Hu, Xingyue Ren, Rowan D. H. Barrett, Eman Samma, Mikolaj Sulkowski, Steven P. Brady

**Affiliations:** ^1^ Ministry of Education Key Laboratory for Biodiversity Science and Ecological Engineering, Center for Evolutionary Biology, School of Life Sciences, Institute of Biodiversity Science Fudan University Shanghai People's Republic of China; ^2^ Department of Biology McGill University Montreal Quebec Canada; ^3^ Biology Department Southern Connecticut State University New Haven Connecticut USA

**Keywords:** alternative splicing, gene expression, local adaptation, maladaptation, population divergence

## Abstract

Phenotypic variation is common across life history and among populations occupying different environments, yet the molecular mechanisms underlying these axes of divergence remain poorly understood. Much work has focused on gene expression as a link between genetic variation, environmental variation, and phenotypes, but post‐transcriptional processes such as alternative splicing—which affect how transcripts are assembled rather than how much of a transcript is produced—are increasingly recognized as additional modulators of plasticity and adaptation. Here, we examined gene expression and alternative splicing together in the wood frog ( *Rana sylvatica* ), an amphibian with a complex life cycle whose populations differ across replicated gradients of road adjacency and associated pollution. We found extensive transcriptomic differences between hatchlings and adults, with thousands of genes differentially expressed or spliced. Individuals clustered strongly by population for both expression and splicing. Differences at the habitat level were less extensive, but revealed two differentially expressed genes (*HSP70* and *Gpsm2*) and one differentially spliced gene (*Cd82*) that consistently distinguished roadside and woodland populations. Overall, genetic differentiation between populations was low, suggesting that phenotypic and transcriptomic differences likely emerge in the presence of gene flow and reflect plastic responses. Together, these results highlight transcriptomic plasticity as an important mechanism shaping variation across both development and population differentiation.

## Graphical abstract

Populations along anthropogenic disturbance gradients exhibit phenotypic differences, yet the mechanisms underlying these variations remain unclear. By examining gene expression and alternative splicing in wood frogs, we found significant life stage and population‐specific transcriptomic differences, though only two genes (*HSP70* and *Gpsm2*) showed repeated divergence between roadside and non‐roadside populations. These results suggest that local adaptation and life history trade‐offs in response to road effects may be driven by complex, non‐parallel transcriptomic mechanisms.

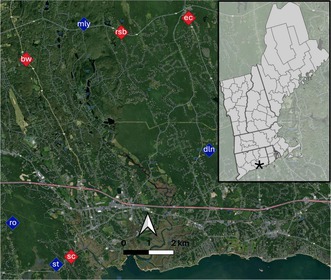

## 1 Introduction

Rapid global change from human activities threatens wild populations across the planet. Pollution driven by urbanization and road expansion continues to create novel and stressful environments for countless biota. Understanding organismal responses to pollution and predicting demographic outcomes remains a core challenge in environmental biology and conservation. Toward this challenge, important insights have been made through envelope‐based approaches (e.g., inferring tolerance to novel or projected conditions) (Hijmans and Graham 2006; Birk et al. 2020) and monitoring‐based approaches (e.g., assessing trends in population size) (Moussy et al. 2022; Morris et al. 2002) focused on trait and population change. Many organisms encounter pollution levels that exceed expected tolerance (Arnott et al. 2020) and indeed many populations are thought to experience declines or even local extinctions due to pollution (Brown et al. 2015; Jepson et al. 2016; Sánchez‐Bayo and Wyckhuys 2019; Henny et al. 2010). At the same time, studies have shown that populations can respond to pollution with adaptive trait plasticity and evolution (Reid et al. 2016; Papadopulos et al. 2021; Brady, Monosson, et al. 2017; Medina et al. 2007; Peña‐Castro et al. 2004). It is increasingly clear that genome‐wide molecular changes underlying plastic and evolved responses to environmental change should be incorporated into projection approaches for more accurate insights and effective management planning (Campbell‐Staton et al. 2020; Salmón et al. 2021; Coffin et al. 2022; Winchell et al. 2023; Chevin et al. 2010; Urban et al. 2016, 2024; Smith et al. 2022). However, the relative importance of plasticity and evolution in shaping adaptive differences between populations inhabiting distinct environments has been less explored (Fox et al. 2019). Thus, knowledge of the molecular drivers of phenotypic variation can strengthen predictive capacity in conservation (Merilä and Hendry 2014; Lu et al. 2024). In other words, predicting the response of organisms to environmental stressors should be well served by studies that aim to link fitness, phenotypes, and genotypes (Barrett and Hoekstra 2011).

Current studies have typically used whole‐transcriptome data as a proxy to explore phenotypic responses in populations experiencing novel environmental conditions (Oleksiak et al. 2002). In contrast to traditional studies that analyze a small number of phenotypic traits (despite many traits being altered by environmental change), the expression level of every gene in the transcriptome can be treated as a phenotypic trait, although not necessarily independent from other traits (Chen and Zhang 2023). In addition, shifts in gene expression are a fundamental cellular response to environmental change, and are widely considered to be important contributors to plasticity and evolution (López‐Maury et al. 2008; Ghalambor et al. 2015; Ho et al. 2020; Fischer et al. 2021). Many studies have successfully identified differentially expressed genes at the transcript level in populations adapted to human‐modified habitats (Coffin et al. 2022; Campbell‐Staton et al. 2021; Wood et al. 2023). However, post‐transcriptional variation underlying organismal responses to anthropogenic disturbance is much less studied than gene expression variation, especially in non‐model species showing local adaptation. Emerging evidence suggests that post‐transcriptional mechanisms such as alternative splicing can play important roles in adaptation (Verta and Jacobs 2021; Singh and Ahi 2022). Unlike changes in transcript abundance, alternative splicing alters exon–intron composition to generate structurally distinct transcripts, providing an independent route for phenotypic change (Wright et al. 2022). Splicing is increasingly recognized as important for both developmental plasticity (Steward et al. 2022a; Tian and Monteiro 2022) and adaptive divergence (Jacobs and Elmer 2021; Healy and Schulte 2019). Despite the importance of transcriptomic variation in regulating phenotypic responses of organisms to environmental change, the roles of gene expression and alternative splicing in facilitating adaptive responses to human‐modified habitats remain largely unexplored. Two fundamental axes of variation may be especially informative: developmental transitions across life history, and differentiation among populations inhabiting contrasting environments. Examining both axes together can help reveal whether the same molecular mechanisms contribute to variation within organisms as they develop and among populations as they differentiate.

Like most organisms on the planet, wild amphibian populations face numerous threats from human activities (Luedtke et al. 2023). Often these threats take the form of novel agents of natural selection capable of imposing strong mortality and reductions in population sizes, and thus a key question in general is whether plasticity and/or adaptive evolution can promote population persistence (Brady and Richardson 2017; Palumbi 2001; Greenspoon and Spencer 2021). Here, our focus is on the effects of road adjacency and runoff pollution on amphibian populations. Across the planet, 40 million paved and unpaved lane kilometers of roads traverse the landscape (Central Intelligence Agency 2022). Mortality caused by vehicle impacts (i.e., ‘roadkill’) is common. Estimates suggest that more than one million vertebrates meet their demise daily, in a real‐life game of frogger. In terms of habitat effects, although roads might be relatively slight in surface area, their ecological effects extend to 20 times their width (Forman and Deblinger 2000). Runoff pollution delivers countless contaminants into adjacent habitats. Hydrocarbons (from fuels, lubricants, and pavement) and heavy metals (from vehicle wear and tear) are notable for their sublethal, toxic, carcinogenic, and even mutagenic effects (Huberman et al. 1976; Kapitulnik et al. 1977). In regions with cold winters, deicing salt is one of the most common runoff pollutants and has caused the salinization of many freshwater habitats and drinking water sources across North America (Dugan et al. 2017; Kelly et al. 2019; Van Meter and Ceisel 2021; Solomon et al. 2023). Numerous studies now report on the myriad impacts of roads and salinization on freshwater organisms, including impacts on population size (Karraker et al. 2008; Hintz and Relyea 2019), life history traits (Zhou et al. 2022; Huang et al. 2022), and physiology (Szeligowski et al. 2022), and there is increasing attention focused on the adaptive potential of populations exposed to these effects (Brady and Richardson 2017; Hintz et al. 2019; Coldsnow et al. 2017; Rogalski and Ferah 2023). Broadly, however, much road salt research has focused on lake and riverine systems. Smaller water bodies like ponds and wetlands that host a unique assemblage of amphibian species have received less attention, yet can reach high salinity, sometimes matching levels found in saltwater estuaries (Brady and Benoit 2025).

The wood frog ( *Rana sylvatica* ) provides a strong system for studying how gene expression and splicing shape different population responses to human‐modified habitats. Our previous work has shown that local populations of wood frogs can differ substantially in tolerance to road adjacency and road salt. Surprisingly, differences in traits between populations at early life history stages contradict predictions for local adaptation. Specifically, through a series of highly replicated field transplant and laboratory exposure experiments (Brady 2013, 2017; Brady, Richardson, and Kunz 2017; Forgione and Brady 2022), we have shown that wood frog embryos from roadside ponds tend to survive at lower rates than their woodland counterparts, whether reared in roadside ponds or in common garden road salt exposure experiments. As larvae, roadside wood frogs also accrue more salt‐induced malformations. Despite this overall survival disadvantage, roadside wood frog populations harbor genetic variation, including genotypes with high embryonic survival in high‐salinity environments (Brady and Goedert 2017). In addition to road salt, roadside ponds typically contain a suite of other runoff contaminants such as plastics, tire particles, hydrocarbons, and heavy metals. Thus, despite maladaptive trait patterns, populations appear to have some capacity for adaptation. Further, because these populations continue to persist, the observed detriments might be the product of tradeoffs across life history stages. Indeed, we have recently found that across aquatic stages, the maladaptive survival disadvantage is limited to embryonic life history stages, beyond which larvae from roadside ponds have rates of survival equivalent to their woodland counterparts, whether reared in common garden road salt exposures or in reciprocal transplant experiments (Forgione and Brady 2022). Thus, any potential tradeoffs countering these negative effects might arise in juveniles or adults. Indeed, gravid females from roadside populations jump further (Brady et al. 2019) and lay more eggs (Brady 2013; Brady et al. 2019) than their woodland counterparts, pointing to several potential tradeoffs. Hereafter, we use the term ‘local (mal)adaptation’ to refer to the uncertainty of whether populations are locally maladapted or locally adapted.

Here, our goal is to begin to understand the molecular basis for (mal)adaptive trait patterns in roadside wood frog populations while simultaneously generating basic insights into gene expression and alternative splicing patterns across life history stages. We use transcriptomic data from a suite of wood frog breeding populations to characterize gene expression and alternative splicing patterns between sexes, between two life history stages (aquatic hatchlings and terrestrial adults), and between populations distributed across a road pollution gradient. Because little is known about wood frog gene expression and alternative splicing patterns in general, we focus on whole hatchlings and several different tissue types (ovary, gonad, unfertilized egg). Our goal with this focus is to provide both broad insights at the organismal level—especially at life history stages when wood frogs experience aquatic pollution—and to generate insights into potential differences across life history stages. We also search for genes that are differentially expressed (DEGs) and/or differentially spliced (DSGs) to identify candidate loci associated with population differentiation between polluted, roadside populations and unpolluted populations located away from roads. Specifically, we ask whether the difference between roadside and woodland populations is predominated by plastic or evolved transcriptomic responses, and if the same gene expression changes are associated with repeated differences between polluted and unpolluted populations, which might help explain previous patterns of local (mal)adaptation.

## 2 Materials and Methods

### 2.1 Natural History and Study Sites

Wood frogs are medium‐sized anurans ranging through much of Canada and the eastern U.S. (Green et al. 2014). Juveniles and adults are terrestrial but often found near ponds used for breeding in spring. Ponds used in this study are located in southern Connecticut, USA, where breeding typically occurs in early March over a period of about 1–2 weeks in a given pond. Adults migrate from uplands to mate in temporary ponds (often referred to as ‘vernal pools’) that tend to dry by late summer. During breeding, males amplex females and fertilize eggs externally upon oviposition. Each female lays an egg mass containing about 800–1100 eggs. Embryos develop over 2–3 weeks before hatching and continue to develop as larvae until metamorphosing into terrestrial juveniles, typically in mid‐summer when they disperse into nearby terrestrial habitats. Adults can live for 5–6 years (Brady et al. 2019; Berven 2009), and apart from the explosive breeding season, tend to spend most of their lives in terrestrial habitats. Each breeding pond is generally considered a population, with low dispersal (13%–20%) typically occurring in the juvenile stage, and almost complete breeding site philopatry among adults (Berven 1990).

**FIGURE 1:**
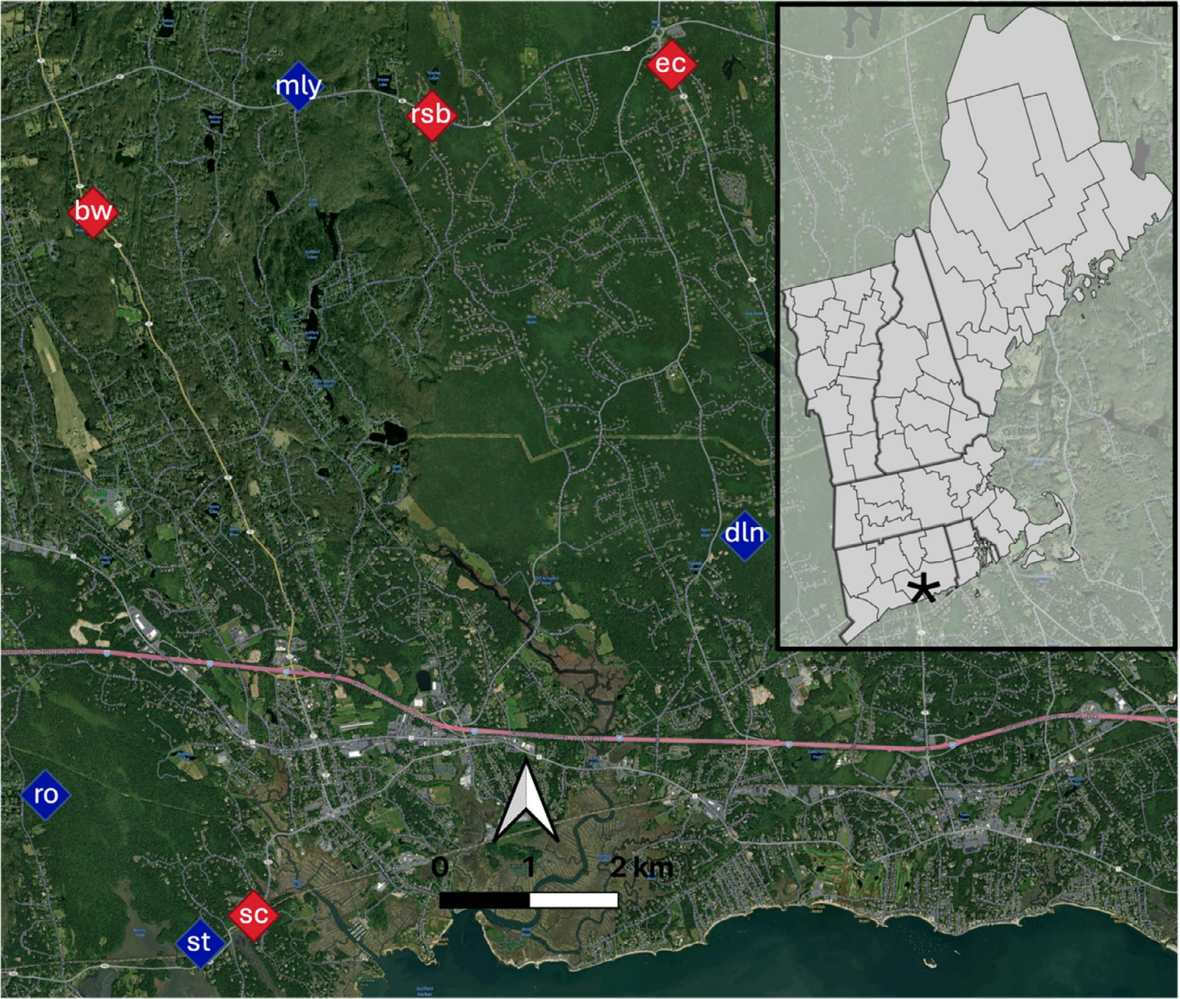
Map of study area and source populations. Roadside ponds are shown in red, woodland ponds are shown in blue. Points are labeled with pond names. Inset shows New England region, with approximate location of study site indicated by asterisk.

We sourced tissue for RNA‐sequencing from wood frogs originating from eight different ponds distributed across a land use gradient (Figure 1). Each pond represents the sampling location for a breeding population. Following our previous approaches (Brady 2013; Brady et al. 2019, 2022), ponds were categorized as either ‘roadside’ (*n* = 4, located < 10 m from a paved road) or ‘woodland’ (*n* = 4, located > 150 m from any road). Distance to the nearest road was measured using leaf‐off satellite imagery from Google Earth. Roadside ponds selected here, like many in our study region, are heavily polluted with road salt, which we typically quantify indirectly using handheld meters that measure specific conductivity (standardized to 25°C). Because background ion concentrations are very low in fresh surface waters in our region, conductivity provides a close proxy for salt pollution. Here, we used a YSI proDDS meter to measure specific conductivity in spring 2022 and 2023 during the early hatchling stages of development. Conductivity was measured at the top and bottom of the water column because roadside ponds often exhibit a vertical salinity gradient (Brady and Benoit 2025). During the wood frog breeding season in spring 2023, the roadside ponds studied here had an average specific conductivity of 625 μS/cm (95% CI: 323.4–926.6) compared to 59 μS/cm (95% CI: 20.1–95.9) in woodland ponds (see Table 1 for study site locations and environmental characteristics). Lab assays of chloride indicate that these specific conductivity values correspond to approximately 150 and 16 mg/L Cl^−^, respectively. Woodland ponds studied here were located within protected areas dominated by large stands of forest and are expected to be generally free of runoff pollutants, based both on previous observations of specific conductivity and on estimates of the distance that chloride from road salt is expected to travel away from roads (Karraker et al. 2008).

**TABLE 1:** Characteristics of sampled ponds and tissues collected for gene expression and splicing.

Pond	Pond type	Tissue(s) collected	Mean conductivity (μS/cm)	Latitude	Longitude	Distance to road (m)
SC	R	Testis (1)	819	41.270905	−72.69239	4
BW	R	Testis (1), ovary (1), unfertilized egg (1), larva (3)	717	41.341658	−72.713874	5
EC	R	Larva (3)	584	41.3565074	−72.636402	7
RSB	R	Larva (3)	380	41.3513833	−72.668501	6
MLY	W	Testis (1)	91	41.3543485	−72.686355	144
ST	W	Testis (1), larva (3)	61	41.2680977	−72.699506	195
RO	W	Larva (3)	44	41.283056	−72.720278	221
DLN	W	Larva (3)	40	41.309122	−72.626594	304

*Note:* Ponds are grouped by type (roadside = R, woodland = W), with those yielding adult tissues listed first in each block. Conductivity values are mean surface conductivity (μS/cm), standardized to 25°C. Tissues collected are listed with sample counts in parentheses. Adult tissue comprised four testes, one ovary, and one unfertilized egg. Hatchlings (larvae at Gosner stage 25) were sampled in groups of three per pond.

### 2.2 Sample Collection

We aimed to generate transcriptomic insights into wood frog gene expression and splicing patterns across populations distributed along an environmental pollution gradient and between life history stages. Given sequencing resource constraints, we prioritized whole hatchling samples to assess population differences, complemented by a subset of adult reproductive tissue samples allocated to examine developmental effects across life history, acknowledging our design limitations (e.g., inter‐cell heterogeneity; Fuess and Bolnick 2023). We initially planned to collect adult and hatchling samples from a suite of four roadside and four woodland ponds, capturing adults during the breeding season and hatchlings from the same sites about 2 months later. However, due to irregular breeding and high subsequent hatchling mortality—likely from the northward expansion of the predator *Ambystoma opacum* —we adapted our sampling approach to ensure adequate representation. This adjustment involved collecting adults from four ponds (*n* = 2 roadside, *n* = 2 woodland) and hatchlings from six ponds (*n* = 3 roadside, *n* = 3 woodland). Among these eight ponds, two ponds used for adult samples did not overlap with the six ponds used for hatchlings. Details of sample origins and tissue types are provided in Table 1.

In March 2023, we used minnow traps to capture one adult male wood frog from each of two roadside and two woodland ponds, and one adult female from one of the roadside ponds. In the laboratory, animals were euthanized via decapitation and pithing to prevent potential impacts of chemical euthanasia on gene expression. Immediately after euthanasia, the abdominal cavity was opened, and the gonads (testes and ovaries) were carefully dissected away from somatic tissue with sterile forceps. Tissues were placed into RNAlater (Thermo Fisher Scientific, Waltham, MA, USA) at room temperature for 2 days before storage at −80°C until RNA extraction.

In late May 2023, hatchling samples were collected from three roadside and three woodland ponds. We used dipnets to collect six hatchlings per pond, sampling individuals from different locations within each pond to minimize the likelihood of collecting siblings. Hatchlings were kept overnight at 10°C in their natal pond water, then viewed under a stereo microscope to score Gosner developmental stage (Gosner 1960) and immediately euthanized by decapitation. Samples were preserved in RNAlater at room temperature for 2 days before storage at −80°C. To standardize developmental effects on gene expression and splicing, we then selected three hatchlings of identical developmental stage (Gosner 25) from each pond (*n* = 18 hatchlings) for whole‐body transcriptomic analysis. In total, 24 samples (*n* = 4 male gonads, *n* = 1 female ovary, *n* = 1 unfertilized egg, and *n* = 18 hatchlings) were shipped on dry ice to BGI America for sequencing. We note that the unfertilized egg sample was included for sequencing in part to determine whether unfertilized eggs could yield suitable transcripts—a potentially valuable insight for future studies of maternal effects.

### 2.3 RNA Extraction and Sequencing

To understand transcriptome‐wide patterns between habitats and life stages, we performed RNA sequencing (RNA‐seq) on all 24 samples. Total RNA was extracted using TRIzol reagent (Invitrogen, Carlsbad, CA, USA), following the manufacturer's protocol. RNA‐seq libraries were constructed by BGI America using the DNBSEQ Eukaryotic Strand‐specific mRNA library preparation protocol. Briefly, Oligo (dT) beads were used to enrich mRNA after DNase I treatment on total RNA, followed by fragmentation and synthesized into first‐strand cDNA. The synthesized cDNA was then purified and ligated to adapters. We selected 250–350 bp fragments for PCR amplification, and the PCR products were then sequenced using 150 bp paired‐end reads. Quality control for total RNA samples, library construction, sequencing, and read cleaning (i.e., adapters and low‐quality reads removal) was all carried out by BGI America.

### 2.4 General and Parallel Gene Expression Variation Analyses

Due to the lack of annotation information for the wood frog reference genome (NCBI accession number GCA_028564925.1), we first used Liftoff v1.6.3 (Shumate and Salzberg 2021) to lift over gene annotation of the common frog ( *Rana temporaria* ) (NCBI accession number GCF_905171775.1) to the wood frog genome, excluding genes with alignment coverage < 20% and sequence identity < 20%. In total, we annotated 26,890 genes in the wood frog reference genome. Following Steward et al. (Steward et al. 2022b), the clean RNA‐seq reads from BGI were aligned to the wood frog reference genome using a two‐step mapping approach implemented in STAR v2.7.10a (Dobin et al. 2013), and only the uniquely mapped reads were retained for downstream analysis. We used the *htseq‐count* function implemented in the Python package HTSeq v0.11.3 (Anders et al. 2015) to quantify read counts for each gene in the wood frog genome.

**FIGURE 2:**
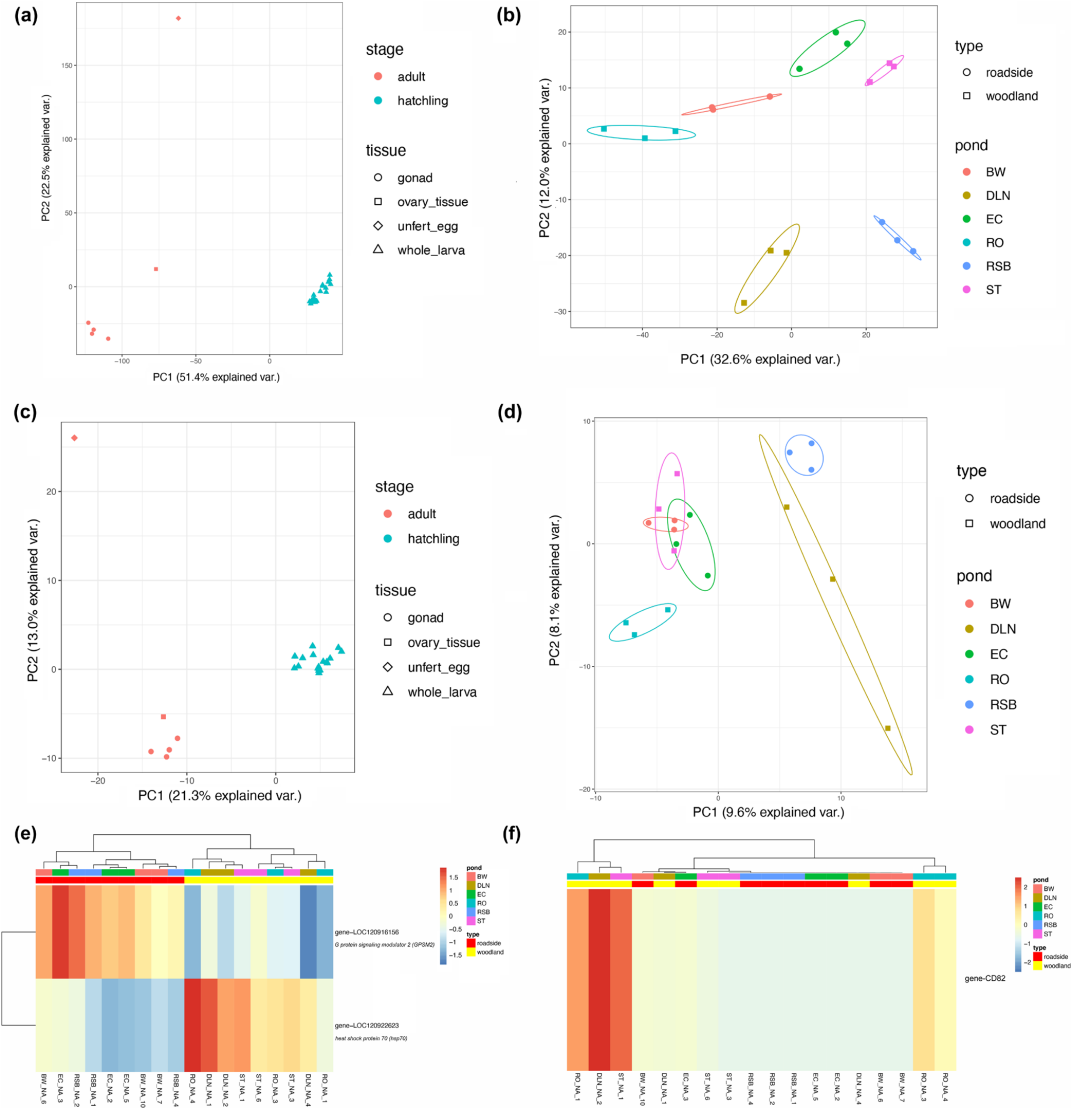
Expression and splicing patterns across life history, tissue, and populations. (a) Principal component analysis (PCA) of gene expression level for a total of 24 adult (*n* = 6, red) and hatchling (*n* = 18, blue) samples, based on *rlog*‐transformed read counts of 12,739 filtered genes. (b) Principal component analysis (PCA) of gene expression level for hatchling samples (*n* = 18) only from six different locations, based on *rlog*‐transformed read counts of 13,554 filtered genes. (c) Principal component analysis (PCA) based on percentage spliced in (PSI) level of all five types of alternative events for a total of 24 adult (*n* = 6, red) and hatchling (*n* = 18, blue) samples. (d) Principal component analysis (PCA) based on percentage spliced in (PSI) level of all five types of alternative events for hatchling samples (*n* = 18) from six different locations. (e) Heatmap of parallel differentially expressed genes (DEGs) in the hatchling samples between roadside and woodland populations (known gene names are labeled on the y‐axis). Each column represents an individual sample. Each row represents one DEG. DEGs and samples are both clustered using the “complete” clustering method based on Euclidean distances of relative gene expression level. The color bar represents the relative gene expression level. Darker red indicates a higher expression level for that DEG in that sample, whereas darker blue indicates a lower expression level. (f) Heatmap of the parallel, differentially spliced gene (DSG) in the hatchling samples between roadside and woodland populations. Each column represents an individual sample. The row represents the DSG (i.e., gene‐*Cd82*). Samples are clustered using the “complete” clustering method based on Euclidean distances of percentage spliced in (PSI) of gene‐*Cd82* reported by rMATS in the alternative splicing event mutually exclusive exons (MXE). Darker red represents higher PSI for the DSG in that sample.

To understand the general expression patterns between samples, we first filtered weakly expressed genes with no expression across all 24 samples, and retained 12,739 genes for downstream analysis. When performing principal component analysis (PCA) on *rlog*‐normalized read counts of the 12,739 genes, we found a clear separation between adult and hatchling samples with an outlier sample of one unfertilized egg (BW_F_113_E, see below) (Figure 2a). We excluded the outlier sample and filtered weakly expressed genes on the remaining 23 libraries, using the same approach described above, and retained 13,554 genes for downstream analysis. For differentially expressed genes (DEGs) analysis, due to the collinearity between tissue and habitat type in the adult samples, we first analyzed gene expression differences between adult (*n* = 5) and hatchling (*n* = 18) samples while controlling for habitat type (design = ~habitat type + stage + habitat type: stage). We identified DEGs using the R package DESeq2 v1.36.9 (Love et al. 2014). We considered genes to be DEGs with Benjamini‐Hochberg adjusted *p*‐values below 0.05. Due to the low sample size of adult samples, we only used hatchling samples for downstream analyses. To further detect DEGs repeatedly showing difference between roadside and woodland populations, we analyzed expression profiles between pairwise roadside‐woodland populations (*n* = 9 pairwise comparisons in total), and excluded expression difference in comparisons of pairwise roadside‐roadside (*n* = 3 pairwise comparisons) and woodland‐woodland (*n* = 3 pairwise comparisons) populations, following an approach similar to Wang et al. (2020). Such a pairwise approach has been suggested to be effective in reducing false positives from habitat‐unrelated differences (Berner and Salzburger 2015). We considered DEGs to be parallel when they were differentially expressed in all roadside‐woodland comparisons, but were not differentially expressed in all roadside‐roadside and woodland‐woodland comparisons. To understand the biological functions of the parallel DEGs, we blasted the sequences of parallel DEGs against standard databases in NCBI. Hit sequences with the highest percent identity and lowest E‐value were regarded as exact matches.

### 2.5 Alternative Splicing Analysis

To investigate alternative splicing patterns between life history stages and habitats, we used rMATS v4.1.0 (Shen et al. 2014) to identify alternative splicing events using the same output files from STAR for the gene expression analysis. rMATS identifies five types of alternative splicing events, including skipped exons (SE), alternative 5′ splice sites (A5SS), alternative 3′ splice sites (A3SS), mutually exclusive exons (MXE), and retained introns (RI). Following Tian and Monteiro (2022), a confident alternative splicing event was identified when a splicing junction had at least 20 counts in the inclusion junctions and at least 20 counts in the skipping junctions across all samples in each comparison. We first performed PCA based on percentage spliced in (PSI) level reported by rMATS for all five types of alternative splicing events across all 24 samples to identify general alternative splicing patterns. Similar to gene expression analysis, we also found the same sample of unfertilized egg to be an outlier, with other samples clustering by their development stages (see below). We thus analyzed splicing differences between life history stages after excluding the outlier sample, and between pooled roadside and pooled woodland hatchling samples. We quantified both annotated and novel splicing junctions to assess differential splicing (DS) events using the same pairwise comparison approach as described above in gene expression analysis, including nine roadside‐woodland comparisons, three woodland‐woodland comparisons, and three roadside‐roadside comparisons. DS events were those with Benjamini‐Hochberg adjusted *p*‐values below 0.05. We considered a gene to be differentially spliced (DSG) when it contained at least one DS event. We considered DSGs to be parallel when they were differentially spliced in all roadside‐woodland comparisons, but were not differentially spliced in all roadside‐roadside and woodland‐woodland comparisons.

### 2.6 Spatial Patterns of Gene Expression and Alternative Splicing Variation

To analyze the spatial patterns of gene expression and splicing variation, we tested the association between expression variation and distance to the nearest road for all populations, as well as the association between splicing variation and distance to the nearest road, using a linear regression approach. We also tested the association between expression variation and the mean conductivity of each sampling site, and the association between splicing variation and the mean conductivity of each sampling site, respectively, using a linear regression approach. We used the first PC from the PCA based on normalized gene expression levels or PSI levels in hatchling samples as the representative of gene expression or alternative splicing variation. We performed the linear regression separately for (1) all hatchlings, (2) roadside hatchlings, and (3) woodland hatchlings. In addition, we calculated pairwise Weir and Cockerham *F*_ST_ (Weir and Cockerham 1984) using vcftools v0.1.16 (Danecek et al. 2011), based on the filtered SNPs extracted from RNA‐seq libraries of all hatchling samples. We filtered SNPs that (1) had a minor allele frequency under 0.005, (2) had more than 10% missing data across all samples, and (3) had high linkage disequilibrium (pairwise *r* ^2^ > 0.8 within a window of 1 Mb). Finally, we performed a PCA based on the filtered SNPs across all hatchling samples to assess the pattern of population differentiation.

## 3 Results

### 3.1 Gene Expression Difference Between Life History Stages and Habitats

Each library consisted of an average of 23,577,070 ± 1,095,917 clean reads, and the average mapping efficiency was 90.0% ± 1.6% (±SD) (ESM Table S1). When performing PCA on all 24 samples, PC1 (variance explained 51.4%) clearly separated adult and hatchling samples, and PC2 clearly separated an outlier sample (unfertilized egg) from both adult and hatchling samples (Figure 2a). When focusing on the hatchling samples, all samples clustered by their ponds with no clear separation between habitats (Figure 2b). Based on the 13,554 genes that passed the filtering step after excluding the outlier sample, we found a large set of 8457 DEGs between adult and hatchling samples while controlling for habitat type. We then focused on the 18 hatchling samples for downstream analysis because of the low statistical power of the adult samples due to the small sample size. In contrast to the large number of DEGs identified between life history stages, we only found 51 DEGs between pooled roadside and pooled woodland samples, among which only two DEGs (i.e., gene‐LOC120916156, gene‐LOC120922623) were found to be repeatedly showing differential expression in all pairwise roadside‐woodland comparisons (Figure 2e), and thus likely to be strong candidates for parallel adaptation. We blasted the two candidate genes against NCBI databases and found that one gene (gene‐LOC120916156) was annotated with *G protein signaling modulator 2* (*Gpsm2*), which was up‐regulated in roadside populations. The other gene (gene‐LOC120922623) was annotated with *heat shock protein 70* (*HSP70*), which was up‐regulated in woodland populations (Figure 2e).

### 3.2 Splicing Variation Between Life History Stages and Habitats

Similar to the general gene expression patterns, the general alternative splicing pattern also showed clear separation between adult and hatchling samples along PC1 (variance explained 21.3%), indicating significant differences between life history stages at the splicing level (Figure 2c). However, we found less separation between hatchling samples from different ponds in splicing compared to gene expression, suggesting relatively higher variance of alternative splicing across habitats (Figure 2d). After excluding the outlier sample (unfertilized egg), we detected 52,643 alternative splicing events in 8175 genes between adult and hatchling samples, among which skipped exons (SE) were the most abundant, whereas retained introns (RI) were the least abundant splicing event. A total of 2343 differentially spliced genes (DSGs) were found between adult and hatchling samples, suggesting extensive changes in splicing between life history stages. Similar to the gene expression analysis above, we focused on the 18 hatchling samples for downstream analysis. When comparing the pooled roadside samples to the pooled woodland samples, we detected 45,435 alternative splicing events in 7559 genes, among which SE were the most abundant, whereas RI were the least abundant splicing event. Although we found a greater number of DSGs (*n* = 473) than differentially expressed genes (DEGs) (*n* = 51) between pooled roadside and pooled woodland samples, only one DSG (*Cd82*) was found to be spliced in parallel in the pairwise comparisons.

### 3.3 Spatial Patterns of Expression and Splicing Variation Are Trivial

**FIGURE 3:**
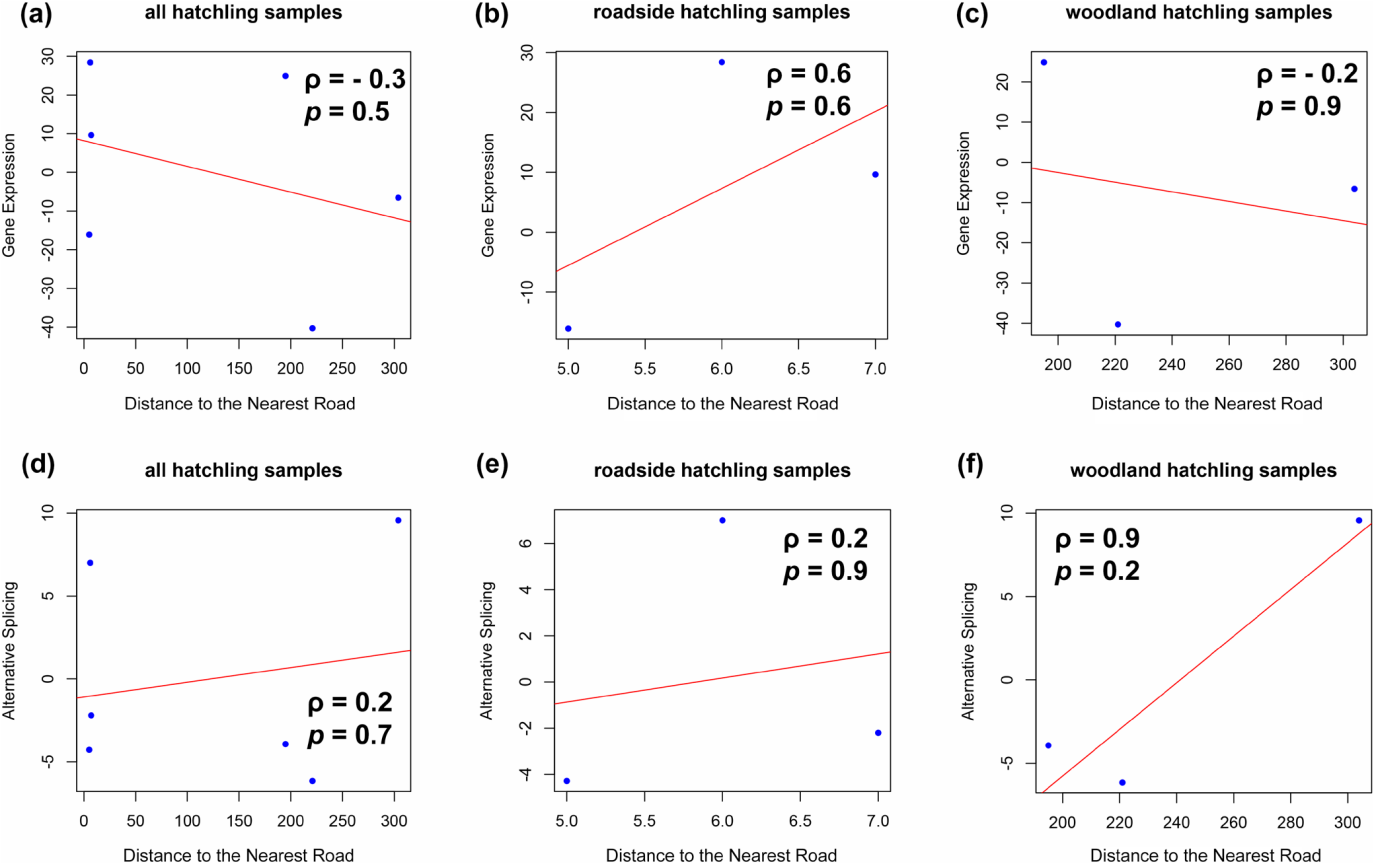
Linear regression for (a–c) gene expression and (d–f) alternative splicing variation against one of the environmental factors: Distance to the nearest road in (a, d) all populations of hatchling samples, (b, e) roadside populations of hatchling samples, and (c, f) woodland populations of hatchling samples.

**FIGURE 4:**
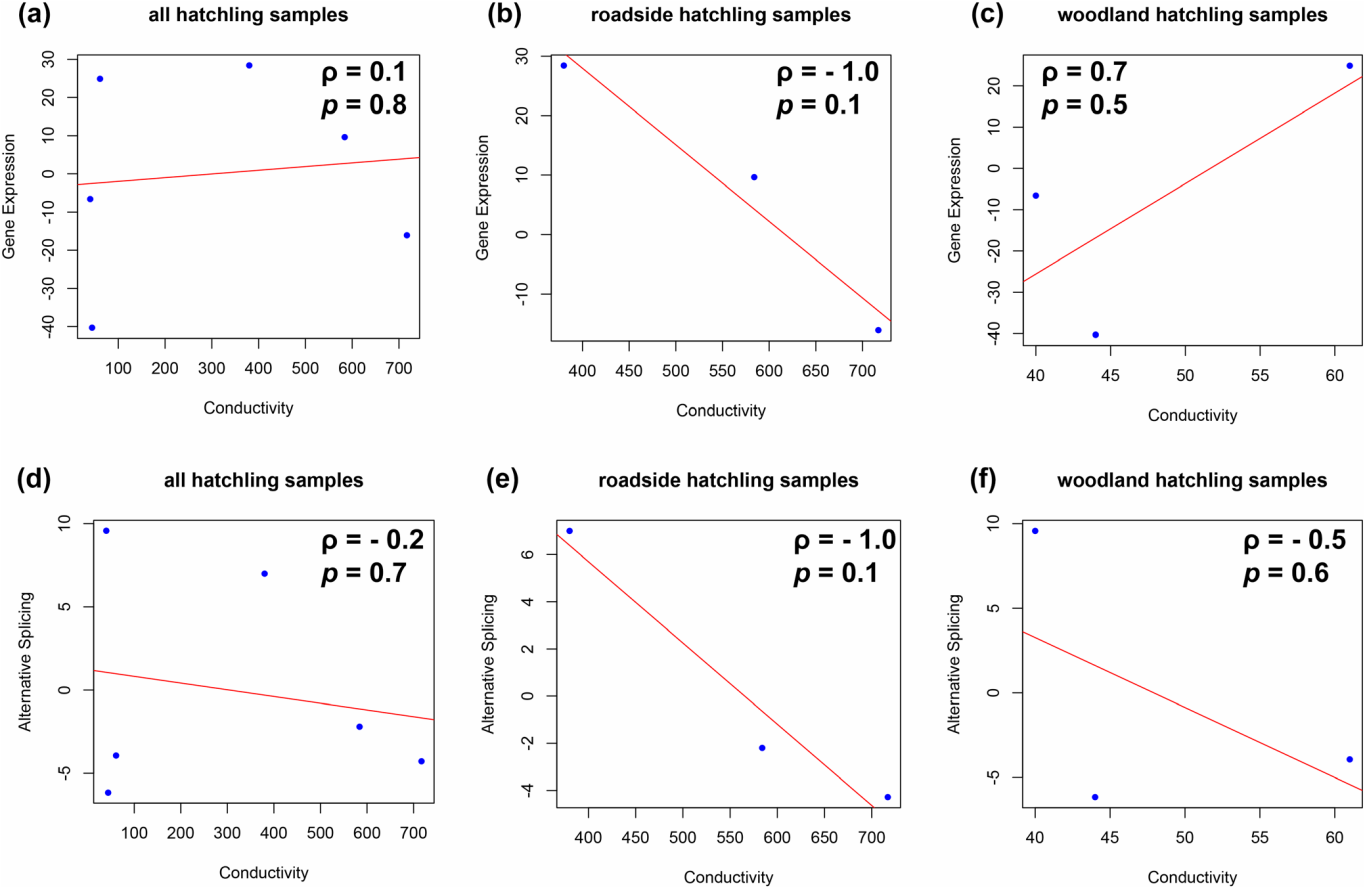
Linear regression for (a–c) gene expression and (d–f) alternative splicing variation against one of the environmental factors: Conductivity in (a, d) all populations of hatchling samples, (b, e) roadside populations of hatchling samples, and (c, f) woodland populations of hatchling samples.

To investigate the spatial patterns of expression and splicing variation, we performed linear regression analyses for all populations of hatchling samples, roadside populations of hatchling samples, and woodland populations of hatchling samples, respectively. We found generally positive but non‐significant associations between distance to the nearest road and both expression and splicing variation (Figure 2). However, the association between the distance to the nearest road and expression in all hatchling samples was negative, possibly due to the strong negative correlation between distance to the nearest road and expression in woodland populations (Figure 3a,c), suggesting distinct responses to pollution between roadside and woodland populations. When analyzing the association between conductivity and both expression and splicing variation, we found that such associations were always in the opposite direction to the associations above in the same group of samples (Figure 4), suggesting that distance to the nearest road and conductivity may represent different aspects of anthropogenic activities that impose distinct impacts on expression and splicing variation patterns. Population differentiation was generally low both within and between habitats [pairwise *F*_ST_ within roadside population: 0.0278 ± 0.0211 (mean ± SD); pairwise *F*_ST_ within woodland population: 0.0332 ± 0.0197 (mean ± SD); pairwise *F*_ST_ across habitat: 0.0301 ± 0.0258 (mean ± SD)]. This result was further supported by the PCA based on the filtered SNPs, which cannot separate between the roadside populations of hatchling samples and the woodland populations of hatchling samples (ESM Figure S1).

## 4 Discussion

Environmental perturbations caused by human activities are driving differentiation between wild populations and can threaten their persistence. Amphibian populations residing in polluted versus unpolluted environments show significant and repeated phenotypic differences (Brady 2013, 2017; Forgione and Brady 2022; Brady and Goedert 2017; Brady et al. 2019), yet we have a limited understanding of the molecular mechanisms underlying these phenotypic changes. Gene expression variation has been strongly implicated in facilitating rapid evolution and development of divergent phenotypes under anthropogenic disturbance (Campbell‐Staton et al. 2020; Coffin et al. 2022; Albecker et al. 2021). While less studied, it has been increasingly acknowledged that alternative splicing can also contribute to phenotypic variation by providing contrasting or redundant routes relative to gene expression (Steward et al. 2022a; Healy and Schulte 2019; Grantham and Brisson 2018). However, the contributions of gene expression and alternative splicing to phenotypic variation due to human activities remain unexplored in amphibians. We quantified gene expression and alternative splicing variation across two axes of variation: (i) between life history stages and (ii) among populations originating from roadside versus woodland habitats. Transcriptomic differences were extensive across development, while population‐level differences were more modest. Fifty‐one genes were differentially expressed between pooled groups of roadside and woodland populations, while 473 genes were differentially spliced. Two genes were divergently expressed, and one gene was divergently spliced, in parallel between all roadside and woodland populations. The relative expression and splicing of these genes point to potential explanations for previously observed local (mal)adaptation patterns discussed below. Together, these results show that gene expression and alternative splicing are highly differentiated across life history stages and between local populations, while three candidate genes identified here might play important roles in shaping repeated phenotypic differences across a land use and pollution gradient. Because only a small number of genes identified here differed in parallel between habitat types, our data suggest the presence of transcriptional flexibility across populations. Thus, nonparallel transcriptional changes might be an important component of phenotypic change for wood frogs in polluted roadside environments (Fischer et al. 2021).

### 4.1 Expression and Splicing Variation Between Life History Stages

PCAs revealed distinct separation of gene expression patterns between hatchlings, one unfertilized egg, and adult reproductive organs (with additional separation between gonad and ovary tissue, although the lack of technical replications for the adult tissues may render relatively low statistical power for us to detect significant differences between these tissues). Collectively, about 62.4% of the 13,554 isolated genes were differentially expressed between hatchlings and adults. Differential expression between life history stages is perhaps not surprising given the high degree of physiological differentiation between pre‐ and post‐metamorphic amphibians. Yet these patterns of variation provide an important backdrop for future inquiry in our study system. Alternative splicing between life history stages follows a comparable pattern to gene expression. A large number of genes (*n* = 7559) produced alternatively spliced isoforms between hatchlings and adults. These results suggest that alternative splicing likely plays an important role in the developmental processes and ultimately the different physiologies of pre‐ and post‐metamorphic amphibians. Indeed, amphibian metamorphosis represents one of the most complex ontogenetic rearrangements of organismal body plans and functions, generally facilitating the transition from obligately aquatic to facultatively terrestrial dwelling. Though morphological changes and underlying endocrine pathways of amphibian metamorphosis have long been identified and studied (Allen 1925; Yoshizato 1990), genetic inquiry remains limited (Brown and Cai 2007), particularly among wild populations. Our study provides the first evidence of splicing differences across amphibian metamorphosis and improves our understanding of the gene regulatory mechanisms occurring during shifts between life history stages.

### 4.2 Expression and Splicing Variation Between Populations

While developmental differences dominated overall transcriptomic variation, we also detected consistent population‐level structuring. Gene expression and (to a lesser extent) alternative splicing differed at the population level, with strong clustering of individuals from the same populations regardless of whether a population originated from a roadside or woodland pond. Thus, each population appears to have its own gene expression and alternative splicing pattern independent of its proximity to roads. This outcome is surprising given the strong phenotypic divergence we have previously documented between roadside and woodland populations, which could be a result of variation in genetic compositions that are not detected in our study or phenotypic plasticity between the two population types. This outcome further suggests that gene expression and splicing structure at the population level might be determined by forces other than pollution, and that there might be diverse routes to achieve adaptation to roadside habitats. For example, other habitat parameters known to influence traits and fitness, such as canopy cover or predation, could be contributing to population structure in gene expression and alternative splicing. However, apart from the effects of road proximity, the ponds we studied here differ in unexpected ways, with gene expression and alternative splicing differences among populations generally showing negative correlation with both geographical distance and environmental factors. Although genetic drift could produce the population structure we observed, the high levels of gene flow inferred between ponds may also explain the non‐significant spatial patterns of gene expression and splicing. This hypothesis is supported by our PCA results, where no clear separation was found between ponds in hatchling samples regardless of habitat type, and is also suggested by the low genetic differentiation between ponds within and between habitat types. Theoretical studies have suggested a migration‐selection balance, where strong gene flow between populations from different environments can cancel the diverging effects of selection (Tigano and Friesen 2016; Barrett and Hoekstra 2011; Savolainen et al. 2013). However, a number of counterexamples, including in amphibians, have shown that adaptive polymorphism can be maintained, and adaptation can still occur despite gene flow (Tepolt et al. 2021; Haenel et al. 2021; Dennenmoser et al. 2017; Jacobs et al. 2024). Indeed, evidence across taxa indicates that microgeographic adaptation is both common and ecologically meaningful, with divergence arising even over fine spatial scales (Richardson et al. 2014). Thus, the equivalent survival rates between roadside and woodland larvae under salinity stress observed in our previous studies may be attributed to the exchange of beneficial alleles between populations to promote persistence under environmental change. Gene flow might also explain the low degree of differentiation between roadside and woodland populations at gene expression and alternative splicing levels (see below), where similar results have also been observed in fish and amphibian species (Wang et al. 2020; Babik et al. 2024). Moreover, our ability to detect differences between woodland and roadside populations may be restricted by low statistical power due to small sample sizes. Nonetheless, our results provide marginal support for one of the interactions (i.e., geographical distance and habitat type for alternative splicing pattern), suggesting that the evolutionary responses might differ between different habitats.

### 4.3 Weak Parallel Patterns of Gene Expression and Splicing Between Roadside and Woodland Habitats

In contrast to the large differences in gene expression and alternative splicing between life history stages, differences between the habitat types were modest. About 0.4% (51 DEGs out of 13,554 filtered genes) of genes were differentially expressed between roadside and woodland habitat types, and about 6.3% (473 DSGs out of 7559 genes with alternative splicing events) were differentially spliced. Specifically, 33 genes were upregulated in roadside populations compared to 18 in woodland populations. In parallel across all roadside–woodland comparisons, two genes were differentially expressed, and one gene was differentially spliced. These findings suggest that while we detected statistically significant differences among habitat types, the differences may not be sufficiently robust to yield consistent and repeated significance in all pairwise comparisons. In addition, the larger number of DSGs than DEGs could possibly be due to the higher flexibility of alternative splicing in regulating responses to environmental change than gene expression (Singh et al. 2017). Crucially, these differences in the expression of these two DEGs and one DSG accord with what might be predicted from patterns of maladaptive aquatic traits and adaptive adult traits in roadside populations, thus providing strong candidate genes to study phenotypic divergence across these populations.

Specifically, *HSP70* was expressed at lower levels in all roadside populations compared to woodland populations. *HSP70* is expressed in virtually every cell in all organisms, functioning as a chaperone to ensure proper folding of newly synthesized proteins and preventing them from forming aggregates (Mayer and Bukau 2005; Tine et al. 2010; Vogel et al. 2006). Generally, *HSP70* is upregulated in response to stressors, including thermal, oxidative, and osmotic stress and exposure to heavy metals (Mahmood et al. 2014; Yusof et al. 2022), where it helps stabilize and refold denatured proteins (Mayer and Bukau 2005). Thus, it seems plausible that the relative downregulation of *HSP70* in polluted roadside habitats contributes to the reduced survival of embryos and increased prevalence of malformations found in these populations and environemnts (Szeligowski et al. 2022; Hintz et al. 2019; Coldsnow et al. 2017; Rogalski and Ferah 2023). Importantly, downregulation of *HSP70* and its presumptive negative effects on embryos and larvae might not necessarily be maladaptive at the population level if it has benefits on other life history stages. Notably, as a molecular chaperone, *HSP70* slows the rate of protein synthesis (Mayer and Bukau 2005). While this post‐translational slowdown, which is part of the unfolded protein response (UPR), maintains cellular function following exposure to adverse conditions, the elevated levels of *HSP70* have been shown to decrease the rate of cell division (Feder et al. 1992). This phenomenon, when extrapolated to the organismal level, could impact growth and reproduction. Consistent with this hypothesis, we have found that exposure to road salt delays metamorphosis (Forgione and Brady 2022) and larval wood frogs reared in roadside ponds have 11.5% lower rates of growth and development compared to those reared in woodland ponds (Brady 2013). Conceivably, these changes might be triggered by an acute upregulation of *HSP70*. Thus, the reduction in *HSP70* transcript in roadside populations may demonstrate a (mal)adaptive response to offset this reproductive slowdown. In support of this idea, adult female wood frogs from roadside populations have rates of fecundity 10.5% higher than woodland females (Brady 2013; Brady et al. 2019). Indeed, gene expression and field studies in the blackchin tilapia (*Sarotherodon melanothero*) have documented an increase in *HSP70* expression correlating with reduced fecundity and saline conditions (Tine et al. 2010; Guèye et al. 2012; Panfili et al. 2004). Thus, we speculate that reduced *HSP70* levels might mediate a tradeoff between fecundity and offspring quality with tolerance to pollution.

In contrast to *HSP70*, *Gpsm2* was expressed at higher levels in roadside compared to woodland populations. *Gpsm2* plays a pivotal role in cellular and developmental processes, and upregulation is linked to increased cancer risk (Zhang et al. 2020; Dang et al. 2021; Hu et al. 2022). Mutations in *Gpsm2* cause the human disease Chudley‐McCullough syndrome (Shahin et al. 2010; Walsh et al. 2010; Doherty et al. 2012), a developmental disorder characterized by sensorineural deafness and a particular set of brain anomalies (agenesis of the corpus callosum, cerebellar dysplasia, and nodular heterotopia). Thus, higher expression in roadside populations might be linked to observed patterns of reduced survival and increased malformations. On the other hand, *Gpsm2* is required for neurite outgrowth, axon guidance, and forms of synaptic plasticity (Doherty et al. 2012; Mauriac et al. 2017; Schiller and Bergstralh 2021). Given these important neurological functions, elevated Gpsm2 levels could potentially explain the increased locomotor performance in roadside populations, whereby gravid females from roadside ponds jump 25% farther than gravid females from woodland ponds (Brady et al. 2019). This variation could stem from roadkill as a source of natural selection, favoring decreased time crossing the road on the route to breeding ponds. Thus, like *HSP70*, *Gpsm2* might be involved in trade‐offs between embryonic/larval and adult traits. These trait trade‐offs might be amplified in roadside ponds if population sizes are smaller, increasing the risk of inbreeding depression and maladaptive larval traits. Indeed, Karraker et al. (2008) demonstrated that road salt exposure can reduce population size dramatically depending on larval density dependence, with more severe declines where compensatory dependence is weak. Nonetheless, in our study system, long‐term observations and prior egg mass surveys indicate that roadside and woodland populations are broadly comparable in abundance and regularly support substantial breeding choruses. Thus, while demographic concerns are plausible, our observations suggest they are unlikely to alone account for the gene expression and splicing patterns we detected.

Notably, recent studies have discovered additional molecular functions for *HSP70*, which accumulates at centrosomes during cell division and is required for mitotic spindle assembly (Fang et al. 2016). It is also required for the formation of pericentriolar material that regulates microtubule dynamics, spindle polarity, and recruitment of regulatory proteins (Fang et al. 2019; Woodruff et al. 2014). Because *Gpsm2* is also a component of the mitotic spindle, an intriguing possibility is that both gene products converge on a common mechanism, i.e., modulating the rate and position of cell division, as part of the complex suite of (mal)adaptive trait divergence found in roadside populations.

Finally, we found one gene (*Cd82*) showing repeated splicing differences between roadside and woodland populations. *Cd82* is a tetraspanin known to regulate cell adhesion, motility, and signaling interactions (Zhou et al. 2010). Alternative isoforms of *Cd82* could influence tissue integrity and developmental processes, potentially contributing to the higher incidence of malformations observed in roadside larvae. Because *Cd82* also interacts with stress and immune pathways, differential splicing at this locus may represent an environmentally responsive mechanism that modulates both development and resilience under polluted conditions.

## 5 Conclusion

Our results contribute to an emerging body of knowledge indicating that alternative splicing and gene expression are important modulators of both developmental transitions and population differentiation. By examining these two axes together, our study highlights transcriptomic plasticity as a central mechanism linking life history shifts with responses to environmental change. The similar patterns found between alternative splicing and gene expression across pre‐ and post‐metamorphic stages of the wood frog should also prompt future studies of alternative splicing in developmental contexts, especially for animals like amphibians with complex life cycles. In conclusion, our study fills an important knowledge gap concerning the roles of gene expression and alternative splicing in regulating amphibian metamorphosis, and helps to reveal the molecular mechanisms by which natural populations respond to road pollution.

## Author Contributions

**Juntao Hu:** conceptualization (equal), data curation (equal), formal analysis (equal), investigation (equal), methodology (equal), project administration (equal), writing – original draft (lead), writing – review and editing (lead). **Xingyue Ren:** data curation (equal), formal analysis (lead), writing – original draft (lead), writing – review and editing (lead). **Rowan D. H. Barrett:** conceptualization (equal), writing – review and editing (equal). **Eman Samma:** data curation (equal), investigation (equal). **Mikolaj Sulkowski:** conceptualization (equal), funding acquisition (equal), writing – original draft (equal), writing – review and editing (equal). **Steven P. Brady:** conceptualization (equal), data curation (equal), funding acquisition (equal), investigation (equal), project administration (equal), writing – original draft (lead), writing – review and editing (lead).

## Conflicts of Interest

The authors declare no conflicts of interest.

## Supporting information


**Figure S1:** ece372481‐sup‐0001‐FigureS1.docx.

**Table S1:** ece372481‐sup‐0001‐TableS1.xlsx.

## Data Availability

Trait and environmental data can be found in Supporting Information. Sequence data are available on NCBI (BioProject accession number: PRJNA1188281), and code is available on GitHub (https://github.com/hulab‐fdu/woodfrog.git).
