# Supplementary material for: Alternative Splicing and Gene Expression Variation Underlie Population and Life History Differences in an Amphibian
Source: Ecol Evol. 2025 Nov 16;15(11):e72481. doi: 10.1002/ece3.72481 (PMC12620059; doi:10.1002/ece3.72481)
Supplement: Supplementary file 1 — Figure S1: ece372481‐sup‐0001‐FigureS1.docx. [file ECE3-15-e72481-s001.docx]

Figure S1 Principal component analysis (PCA) based on all 216,545 filtered SNPs in hatchling samples.
